# The simplified trachoma grading system, amended

**DOI:** 10.2471/BLT.19.248708

**Published:** 2020-09-03

**Authors:** Anthony W Solomon, Amir B Kello, Mathieu Bangert, Sheila K West, Hugh R Taylor, Rabebe Tekeraoi, Allen Foster

**Affiliations:** a Department of Control of Neglected Tropical Diseases, World Health Organization, Avenue Appia 20, 1211 Geneva 27, Switzerland.; b World Health Organization Regional Office for Africa, Brazzaville, Congo.; c Wilmer Eye Institute, Johns Hopkins University, Baltimore, United States of America.; d Melbourne School of Population and Global Health, University of Melbourne, Melbourne, Australia.; e Eye Department, Ministry of Health and Medical Services, South Tarawa, Kiribati.; f International Centre for Eye Health, London School of Hygiene & Tropical Medicine, London, England.

## Abstract

A simplified grading system for trachoma was published by the World Health Organization (WHO) in 1987. Intended for use by non-specialist personnel working at community level, the system includes five signs, each of which can be present or absent in any eye: (i) trachomatous trichiasis; (ii) corneal opacity; (iii) trachomatous inflammation—follicular; (iv) trachomatous inflammation—intense; and (v) trachomatous scarring. Though neither perfectly sensitive nor perfectly specific for trachoma, these signs have been essential tools for identifying populations that need interventions to eliminate trachoma as a public health problem. In 2018, at WHO’s 4th global scientific meeting on trachoma, the definition of one of the signs, trachomatous trichiasis, was amended to exclude trichiasis that affects only the lower eyelid. This paper presents the amended system, updates its presentation, offers notes on its use and identifies areas of ongoing debate.

## Résumé

En 1987, l'Organisation mondiale de la Santé a publié un système de codage simplifié du trachome. Destiné au personnel non qualifié travaillant au sein des communautés, il comporte cinq signes, chacun pouvant être présent ou absent dans l'un ou l'autre œil: (i) le trichiasis trachomateux; (ii) l'opacité cornéenne; (iii) l'inflammation trachomateuse — folliculaire; (iv) l'inflammation trachomateuse — intense; et enfin, (v) la cicatrice trachomateuse. Bien qu'ils ne soient ni parfaitement précis, ni totalement spécifiques au trachome, ces signes constituent des outils essentiels pour identifier les populations qui nécessitent une intervention afin d'éliminer le trachome en tant que problème de santé publique. En 2018, lors de la quatrième réunion scientifique mondiale sur le trachome, la définition de l'un des signes, le trichiasis trachomateux, a été modifiée pour exclure du système de codage le trichiasis qui n'affecte que la paupière inférieure. Ce document expose le nouveau système, actualise sa présentation, formule des remarques sur son utilisation et identifie les domaines qui font encore l'objet de débats.

## Resumen

En 1987, la Organización Mundial de la Salud (OMS) publicó un sistema de clasificación simplificado para el tracoma. Este sistema fue diseñado para que lo utilice el personal no especializado que trabaja a nivel comunitario e incluye cinco signos, cada uno de los cuales puede estar presente o ausente en los ojos: i) la triquiasis tracomatosa; ii) la opacidad corneal; iii) la inflamación tracomatosa-folicular; iv) la inflamación tracomatosa-intensa; y v) la cicatrización tracomatosa. Si bien no son perfectamente sensibles ni muy específicos del tracoma, estos signos han sido herramientas esenciales para identificar a las poblaciones que requieren intervenciones para eliminar el tracoma como problema de salud pública. En 2018, se modificó la definición de uno de los signos, la triquiasis tracomatosa, en la 4.ª Reunión Científica Mundial sobre el Tracoma de la OMS, para descartar la triquiasis que solo afecta al párpado inferior. En el presente documento se describe el sistema modificado, se actualiza su presentación, se ofrecen observaciones sobre su aplicación y se identifican los ámbitos de debate en curso.

## ملخص

تم نشر نظام تصنيف مبسط للتراكوما من جانب منظمة الصحة العالمية (WHO) في عام 1987. وهو مخصص للاستخدام بواسطة الأشخاص غير المتخصصين الذين يعملون على مستوى المجتمع، ويشمل النظام خمس علامات يمكن أن يكون كل منها موجودًا أو غير موجود في أي عين: (1) داء الشعرة التراكومي؛ و(2) عتامة القرنية؛ و(3) الالتهاب الجريبي التراكومي؛ و(4) الالتهاب التراكومي الشديد؛ و(5) التندب التراكومي. وبالرغم من أن هذه العلامات لم تكن حساسة للغاية أو محددة للإصابة بالتراكوما، إلا أنها كانت مؤشرات أساسية لتحديد السكان الذين يحتاجون إلى تدخلات طبية للقضاء على التراكوما كمشكلة صحية عامة. في عام 2018، في الاجتماع العلمي العالمي الرابع لمنظمة الصحة العالمية حول التراكوما، تم تعديل تعريف إحدى العلامات، وهو داء الشعرة التراكومي، وذلك لاستبعاد داء الشعرة الذي يصيب الجفن السفلي فقط. تعرض هذه الورقة النظام المعدل، وتقوم بتحديث عرضه التقديمي، وتقدم ملاحظات حول استخدامه، وتحدد مجالات النقاش الدائر.

## 摘要

1987 年,世界卫生组织 (WHO) 公布了沙眼简化分级系统。该系统旨在供社区非专业工作人员使用，具备五种体征，其中每个体征都可出现于任一眼睛中，也可能不出现：(I) 沙眼性倒睫；(ii) 角膜混浊； (iii) 沙眼性炎症-滤泡； (iv) 沙眼性剧烈-炎症；以及 (v) 沙眼性疤痕。尽管对沙眼而言，这些体征即非特别敏感，也非专属于沙眼，但其已是确定哪些民众需通过干预消除沙眼这个公共卫生问题的关键。2018 年世卫组织第四届全球沙眼科学会议对沙眼性倒睫的定义进行了修正，排除了仅影响下眼睑的倒睫。本文介绍了修正后的系统，并更新了其介绍，给出了使用说明，并确定了正在讨论中的领域。

## Резюме

В 1987 году Всемирная организация здравоохранения (ВОЗ) опубликовала упрощенную систему оценки трахомы. Предназначенная для использования неспециализированным персоналом, работающим на местном уровне, система включает пять признаков, каждый из которых может присутствовать или отсутствовать в любом глазу: (i) трахоматозный трихиаз; (ii) помутнение роговицы; (iii) трахоматозное воспаление фолликулярное; (iv) трахоматозное воспаление интенсивное; (v) трахоматозное рубцевание. Хотя эти признаки не являются ни абсолютно чувствительными, ни абсолютно специфичными для трахомы, они были важными инструментами для выявления групп населения, которым необходимы вмешательства для устранения трахомы как проблемы общественного здравоохранения. В 2018 году на 4-й Всемирной научной конференции ВОЗ по вопросам трахомы определение одного из признаков, трахоматозного трихиаза, было изменено, чтобы исключить трихиаз, поражающий только нижнее веко. В данном документе приведена измененная система, обновлено ее представление, даны примечания по ее использованию и определены сферы текущих дискуссий.

## Introduction

Trachoma is the most important infectious cause of blindness.^1^ Repeated conjunctival infection^2^ with particular strains of *Chlamydia trachomatis*^3^^–^^5^ results, in some people, in conjunctival scarring, trichiasis and corneal opacity. The disease is strongly linked to poverty.^6^ In March 2019, 142 million people globally were at risk.^7^

At the population level, trachoma may be hyperendemic, severe and blinding; or less severe and non-blinding.^8^ Models suggest that more than 150 episodes of *C. trachomatis* infection are required to develop trichiasis from trachoma;^9^ immune responses to infection are critical in the pathogenetic pathway.^10^^,^^11^ Populations therefore presumably transition from having blinding to non-blinding disease through reductions in intensity of ocular *C. trachomatis* transmission. These reductions in transmission can occur through general socioeconomic improvement^12^^,^^13^ or specific implementation of the antibiotics, facial cleanliness and environmental improvement components of the SAFE strategy for trachoma elimination;^14^ the S of SAFE representing surgery for trichiasis,^15^ which does not alter transmission. However, *C. trachomatis* transmission intensity^16^^,^^17^ would be extremely difficult to measure directly. In contrast, conjunctival inflammatory responses to infection^11^ can be observed by examination of the conjunctiva under low-power magnification. Because of this, and because the vision-impairing effects of trachoma are manifest through macroscopic changes in the eyelid and cornea, assessment of the public health impact of trachoma is carried out through detection of physical signs of disease.^18^

In 1987, the World Health Organization (WHO) published the simplified trachoma grading system to provide a standardized method for non-specialist personnel to undertake such assessments.^19^ The system, which was field-tested in three countries,^19^^,^^20^ has five signs: (i) trachomatous trichiasis; (ii) corneal opacity; (iii) trachomatous inflammation—follicular; (iv) trachomatous inflammation—intense; and (v) trachomatous scarring.^19^ Trachomatous inflammation—follicular and trachomatous inflammation—intense are signs of active (inflammatory) trachoma, usually associated with conjunctival *C. trachomatis* infection.^21^ The signs of the simplified system have been used to complete baseline mapping of trachoma in suspected endemic districts worldwide,^22^^,^^23^ to assess the impact of interventions in both research^24^^,^^25^ and programme^26^^,^^27^ contexts, and to establish criteria for elimination of trachoma as a public health problem.^28^

During recent programmatic scale-up and transition of countries through the validation process,^29^ technical questions have arisen about the use of some of the simplified system’s signs. In November 2018, WHO convened the 4th global scientific meeting on trachoma in part to address those questions. In particular, the meeting considered whether the prevalence of trachomatous inflammation—follicular in 1–9-year-olds remains the best way for programmes to determine whether ocular *C. trachomatis* transmission intensity has been sufficiently reduced to minimize the risk of future trachomatous blindness. Also considered was whether the definition of trachomatous trichiasis could be improved to better differentiate trachomatous from non-trachomatous trichiasis. As a consequence, the definition of trachomatous trichiasis was amended to exclude trichiasis affecting only the lower eyelid, on the basis that such cases are unlikely to be due to trachoma and that labelling them as trachomatous trichiasis constitutes potential misclassification.^30^

In the current paper we set out the simplified system including that amendment, and revise the presentation of the system to help users better understand and more consistently apply the definitions. We discuss the continuing programmatic dependence on the prevalence of trachomatous inflammation—follicular as a clinical proxy for current ocular *C. trachomatis* transmission intensity. Finally, we outline other topics for continuing research and reflection. Inevitably, this paper draws heavily from the original article.^19^

## Method of examination

The examiner should always use clean hands (by, for example, applying alcohol-based hand sanitizer), binocular loupes (magnification × 2.5) and adequate lighting (either sunlight or a torch). Although sunlight provides the best illumination for examining the conjunctiva, it is better to examine for trachomatous trichiasis indoors because of the photophobia that patients with trichiasis experience. An examination station on the edge of a shaded area can be ideal, but survey teams typically move house-to-house. When examining the conjunctiva outdoors, it is important to orient the person being examined so that light from the sun passes across the examiner’s shoulder onto the person’s face. When using a torch, care should be taken not to contaminate it with fingers that have been in contact with the person’s skin or eye. An assistant can help to hold and reassure children while they are examined.^31^

To help ensure that abnormal findings are correctly recorded, the right eye should always be examined first, then the left eye. To assess for the presence or absence of each of the signs of the system, the structures to be examined in each eye are, in order: the upper eyelid margin and its eyelashes, then the cornea, then the upper tarsal conjunctiva (the conjunctiva lining the upper eyelid).

To examine for trachomatous trichiasis, with the examinee’s eye open and the eyelid in its resting state, the examiner should adopt a position so that his or her eyes are below the horizontal plane through the person’s eye. Light should be shone from below then from the temporal side while the examiner inspects the upper eyelid eyelashes to see if any eyelashes touch the eyeball. The examinee should be asked to move their eyes to the extremes of gaze on either side to see if the upper eyelid eyelashes move with the eyeball. The upper eyelid should then be gently lifted upwards slightly to expose the eyelid margin and lift any in-turned eyelashes out of the tear film. The eyelid margin should be inspected for empty eyelash follicles or broken eyelash shafts, which may indicate recent epilation.

The cornea should be inspected for opacities.

**Fig. 1:**
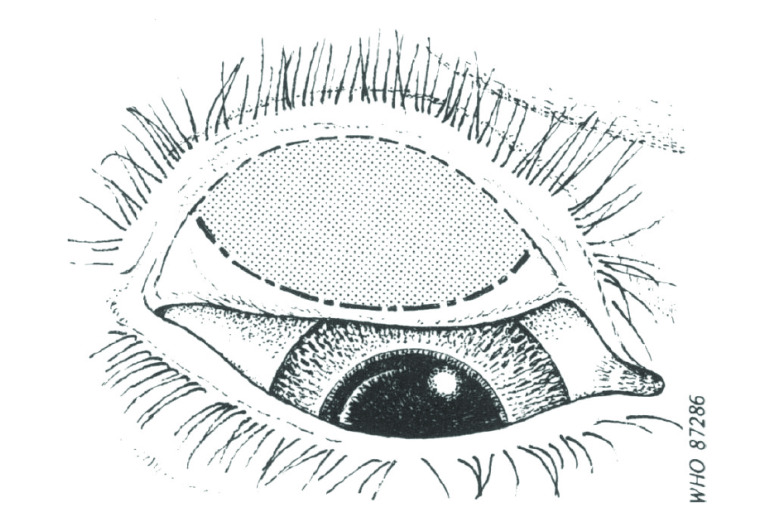
Outline sketch of an everted upper eyelid

Note: The drawing shows the area of the tarsal conjunctiva (shaded) to be examined for follicles.

The eyelid is everted and the conjunctiva inspected for follicles, pronounced inflammatory thickening, and scarring. When examining for follicles, only those found within the central part of the upper tarsal conjunctiva (Fig. 1) are considered significant.^19^

## Appearance of the normal eye

**Fig. 2:**
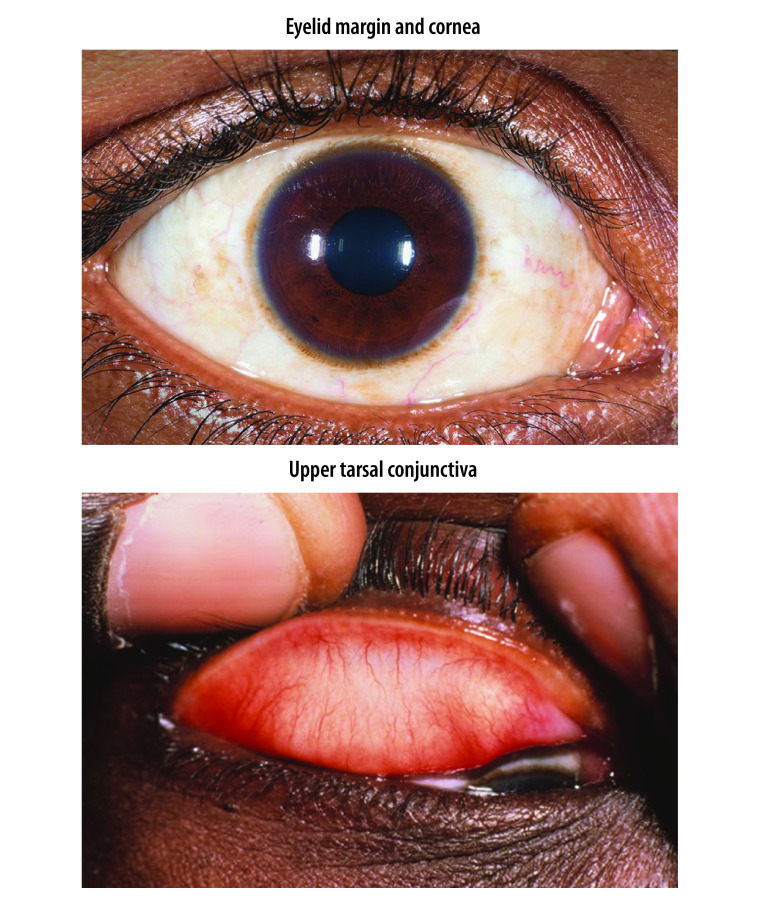
A normal eye

The appearance of the normal eye when examined for trachoma is as follows (Fig. 2): (i) no eyelashes touch the eyeball and there is no evidence of recent epilation; (ii) the cornea is smooth, transparent and avascular; and (iii) the upper tarsal conjunctiva is smooth, thin and transparent. Beneath the whole area of the upper tarsal conjunctiva, there are large deep-lying blood vessels that mainly run vertically from the upper and lower edges of the tarsal plate.^19^

Credit: Sandip Das Sanyam

## Amended simplified grading system

**Fig. 3:**
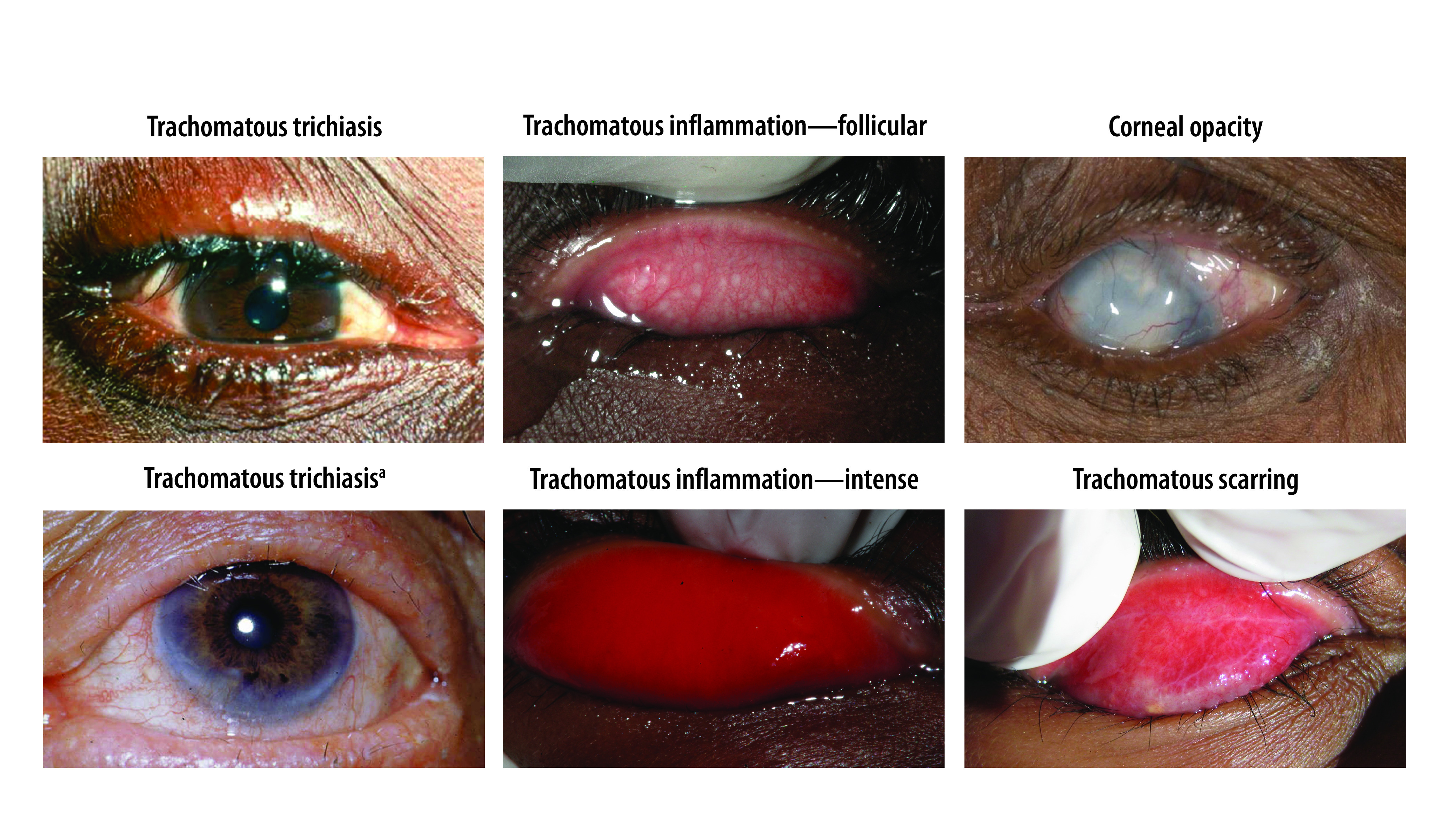
The five signs of the World Health Organization simplified trachoma grading system

^a ^Trachomatous trichiasis manifest through evidence of recent epilation of in-turned eyelashes from the upper eyelid.

The amended simplified grading system includes five signs, each of which can be present or absent. A sign must be clearly seen to be considered present. If the examiner is in doubt, a sign should be recorded as being absent. The list below presents the definitions of the signs. Amendments from the original grading system^19^ are shown in italics (Fig. 3).

### Trachomatous trichiasis

At least one eyelash *from the upper eyelid touches* the eyeball, or evidence of recent epilation of in-turned eyelashes *from the upper eyelid*. 

### Corneal opacity

Easily visible corneal opacity *that is so dense that at least part* of the pupil *margin is blurred when viewed through the opacity*. This definition is intended to detect corneal opacities that cause significant visual impairment. In individuals with corneal opacity, visual acuity should be measured, if possible.

### Trachomatous inflammation—follicular

The presence of five or more follicles, *each at least 0.5 mm in diameter,* in the *central part of the* upper tarsal conjunctiva. Follicles are round lumps or spots that lie beneath more superficial epithelium and are paler than the surrounding tissue. Care should be taken to distinguish follicles from small scars, conjunctival concretions, cysts and giant papillae (please see below). 

### Trachomatous inflammation—intense

Pronounced inflammatory thickening of the upper tarsal conjunctiva that obscures more than half of the normal deep tarsal vessels. The key feature of trachomatous inflammation—intense is pronounced inflammatory *thickening*, which is defined as being present when more than half of the normal deep tarsal vessels are not visible because they are obscured by inflammatory infiltration. The tarsal conjunctiva appears red, rough and thickened, due to diffuse infiltration, oedema and enlargement of vascular tufts (papillary hypertrophy). There are also usually numerous follicles, which may be partially or totally obscured by the thickened conjunctiva. Inflammatory thickening and opacification of the conjunctiva should not be confused with that caused by scarring, which can manifest as diffuse fibrosis or formation of a fibrovascular membrane.

### Trachomatous scarring

The presence of *easily visible* scarring in the *upper* tarsal conjunctiva. Scars are white lines, bands, or sheets in the upper tarsal conjunctiva. Characteristically, the scars are glistening and fibrous in appearance, with straight, angular or feathered edges. Scarring, especially diffuse fibrosis, may obscure tarsal blood vessels, but this must not be confused with diffuse inflammatory thickening.

## Less florid presentations

Some eyes do not have signs of trachoma as defined above but are nevertheless abnormal. For example, the upper tarsal conjunctiva may have any of the following: from one to four central follicles each at least 0.5 mm in diameter; five or more central follicles but fewer than five that are at least 0.5 mm in diameter; inflammatory thickening that does not obscure half of the deep tarsal vessels; more than half of the deep tarsal vessels obscured by something other than pronounced inflammatory thickening. 

Eyes should not be recorded as having a sign whose definition they do not meet. Graders should be confident that this is not equivalent to saying that they are normal eyes.

## Other abnormalities

**Fig. 4:**
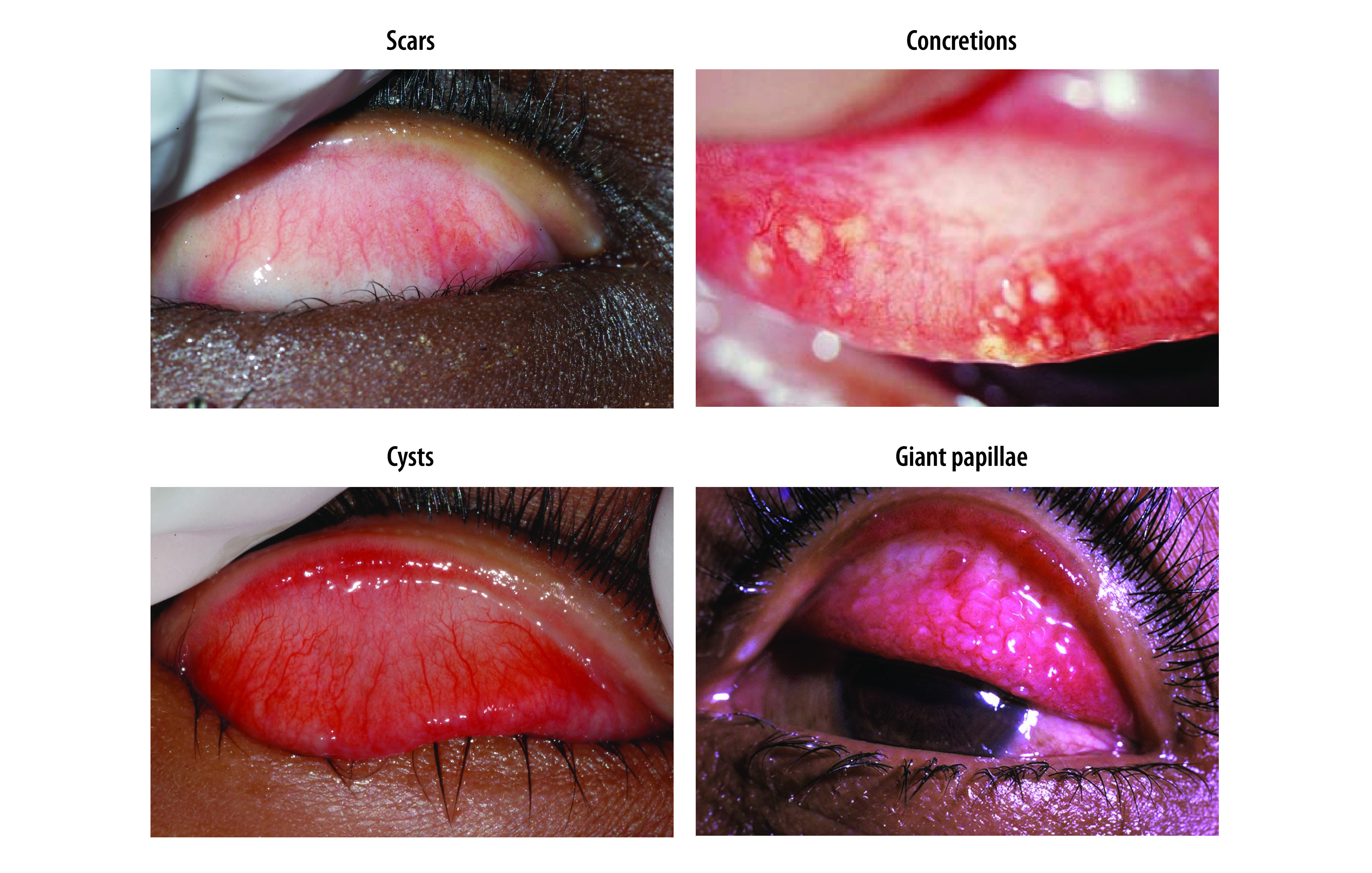
Abnormalities of the conjunctiva that are sometimes mistaken for follicles

Notes: Credit to Thomas M Lietman for the cysts photo and to Jock Anderson for the giant papillae photo.

Other abnormalities that can be mistaken for follicles are illustrated in Fig. 4. Scars have angular borders with sharp corners, whereas follicles have rounded edges that are not sharply defined. Concretions are yellow or white masses with clear-cut edges. Cysts are clear bubbles in the conjunctiva. Giant papillae are protuberances of the conjunctival epithelium that are greater than 1 mm in diameter; they characteristically have flattened tops and are responsible for more pronounced elevation of the epithelial surface than occurs with follicles.

## Discussion

Clear definitions are a prerequisite for collecting good data. Broad consensus on the relevance of the data being collected increases the likelihood that the outputs of the data collection process will be accepted and applied.^32^ The amendment to the definition of trachomatous trichiasis introduced in this version of the WHO simplified trachoma grading system has been endorsed by WHO. This endorsement follows the consensus, reached within an expert panel,^30^ that trachomatous trichiasis characteristically affects upper eyelids, with additional involvement of lower eyelids in a minority of people. Involutional entropion, an age-related condition, does not affect upper eyelids. Excluding lower-eyelid-only disease will avoid trichiasis caused by involutional entropion being misinterpreted as trachomatous trichiasis.

In addition to recording the amended definition of trachomatous trichiasis, we have attempted in this paper to aid users of the simplified system through changes that streamline and clarify its presentation. Our changes are in line with other recent efforts to improve quality control and quality assurance in the assessment of trachoma for public health purposes.^33^

First, we have separated the description of the normal eye from the presentation of the signs of the system. In the original paper, under the heading “The grading system and its use,” the first subheading was “Normal conjunctiva” followed by subheadings for each of the five signs.^19^ This layout may have encouraged the (unfortunately frequent) misunderstandings that normal conjunctiva and the five signs of the system are points along a continuum of disease, mutually exclusive, or an all-encompassing classification system. Trachoma involves repeated cycles of conjunctival *C. trachomatis* infection, active trachoma and resolution, driving an underlying scarring process.^2^^,^^9^ An eye may have any combination of signs present at the same time; and an eye may demonstrate evidence of active trachoma that is insufficiently florid to meet the definition of either trachomatous inflammation—follicular or trachomatous inflammation—intense, trachomatous conjunctival scar that is insufficiently severe to meet the definition of trachomatous scarring, or a trachomatous corneal scar that is insufficiently dense or too close to the limbus to meet the definition of corneal opacity. There may also be signs of trachoma not mentioned in the system,^34^ or abnormalities unrelated to trachoma.

Second, we have tried to ensure that all the critical elements of the definition of each sign are included in the formal definition statement. For example, for trachomatous inflammation—follicular, we have included in the statement the requirements for follicles to be greater than or equal to *0.5 mm in diameter* and located in *the central part of* the upper tarsal conjunctiva. Previously the statement read, “the presence of five or more follicles in the upper tarsal conjunctiva,” with notes on the area to be examined and the minimum follicle size included elsewhere in the text of the paper.^19^ These refinements should facilitate use of the grading system.

Third, we have placed the definitions for trachomatous trichiasis and corneal opacity above those for trachomatous inflammation—follicular, trachomatous inflammation—intense and trachomatous scarring. When conducting a physical examination, the general principle is to look before touching, and to undertake less invasive before more invasive examination steps. Examination for the five signs of the trachoma grading system, therefore, should commence with examination for trachomatous trichiasis, then examination for corneal opacity, then lid eversion and examination of the conjunctiva. This sequence was recommended within the text of the original paper, but the order in which the signs were presented appeared to contradict it.^19^ The revised order will also hopefully help to counteract the common but incorrect impression that the signs listed in the simplified grading system develop in inevitable sequential order after acquisition of ocular *C. trachomatis* infection.

Fourth, unlike the original paper, the new presentation of the grading system does not support discretionary use of “loupes of higher magnification or a slit lamp.”^19^ The rationale for removing this option is straightforward. Slit lamps are rarely available for field use in trachoma surveys. Standardization of examination methods is important to maximize their reproducibility.^33^ Corneal opacity and conjunctival scarring must be easily visible to be graded as corneal opacity and trachomatous scarring, respectively. If magnification and illumination differ between examiners or between time points, what is easily visible in one instance may not be easily visible in another.

It is worth emphasizing here that the grading system was designed, and is still principally intended, for non-specialist health personnel working in the community.^19^ The grading system is an essential tool for population-based surveys, where large numbers of graders need to be trained to generate accurate data on trachomatous trichiasis and trachomatous inflammation—follicular, the signs most directly linked to programmatic action and which are used in the criteria for the elimination of trachoma as a public health problem.^28^ As noted above, many individuals have clear evidence of trachoma but do not have disease meeting the definition for any of the five signs codified here. More detailed systems, such as the modified WHO FPC system (follicles, papillary hypertrophy and diffuse infiltration, conjunctival scarring),^34^ may therefore be more suitable for other applications, particularly research studies.^35^ Use of a more detailed grading system may be important in studies using highly sophisticated laboratory tests, such as polymerase chain reaction testing, in which the results of the tests are compared with the presence of signs at the individual level.^36^^,^^37^

Research into and discussion of further refinement of the simplified trachoma grading system would be welcome. For trachomatous trichiasis, we believe that two components of the amended definition deserve ongoing attention.

First, “evidence of recent removal of in-turned eyelashes” qualifies an eye to be diagnosed with trachomatous trichiasis. This definition implies that all epilated eyelashes were in-turned, although data suggest that field graders may have difficulty determining whether or not an epilated eyelash was in-turned before it was pulled out.^38^ The meaning of “recent” within this context has never been defined, but has been interpreted by users of the system as the appearance of broken eyelashes or empty eyelash follicles.

Second, data are needed to determine whether restriction of diagnostic focus to the upper eyelid is sufficient to exclude, as intended, most cases of non-trachomatous trichiasis. This point is essential for trachoma programmes, which for the purposes of validating elimination of trachoma as a public health problem must demonstrate the trachomatous trichiasis prevalence unknown to the health system in individuals aged 15 years and older is less than 0.2%. Trichiasis can be caused by processes other than trachoma, and the background prevalence of non-trachomatous trichiasis is usually not known for any particular area. There is ongoing research into whether the presence of trachomatous scarring (or conjunctival scar more generally) should be a requirement for trichiasis to be considered due to trachoma, as discussed at the Second Global Scientific Meeting on Trachomatous Trichiasis.^39^ Such an approach would be consistent with earlier, more detailed WHO trachoma grading systems.^40^^,^^41^ These systems included trichiasis wholly within the diagnostic domains for conjunctival scarring, implying that, in trachoma, trichiasis was exclusively an end-stage feature of a scarred eyelid. Similarly, the original presentation of the simplified system noted that, “for the potentially disabling, irreversible lesions, trachomatous scarring may represent all levels of conjunctival scarring, whereas the coding of trachomatous trichiasis corresponds to more severe cases.”^19^ Some authorities, however, are concerned about the repeatability, sensitivity or specificity of the diagnosis of trachomatous scarring.^30^ An alternative algorithm might involve reference, at individual or evaluation unit level, to the presence of corneal pannus at the upper pole of the limbus or to Herbert’s pits,^42^ each of which is considered highly specific for trachoma. Unfortunately, these signs are also likely to be imperfectly sensitive.

For trachomatous inflammation—follicular, the size of the follicles matters, although there are no experimental data to show that follicles of different sizes produce differential risk of scarring. The requirement that follicles should be at least 0.5 mm in diameter was introduced more than three decades ago. The aim of the change then was to improve inter-observer agreement, which had been suboptimal when using a draft definition of trachomatous inflammation—follicular in initial tests of the system.^19^ Unfortunately, the line drawing of trachomatous inflammation—follicular that was included in the original simplified system paper^19^ displayed follicles which (when calibrated with reference to photographs of the everted conjunctivae of children from Ethiopia and Niger) have a diameter 20% smaller than 0.5 mm.^43^ We have omitted the diagram from this paper. Follicle size guides^44^ have recently been introduced to help graders to consistently diagnose the presence or absence of trachomatous inflammation—follicular according to the agreed definition.

A separate concern for trachomatous inflammation—follicular in some settings is the extent to which its prevalence may give a false impression of ocular *C. trachomatis* infection or the intensity of ocular *C. trachomatis* transmission.^45^^–^^50^ More investigation on this is needed.^30^

The global prevalence of trachoma is progressively declining,^7^ due to a combination of intensive programmatic intervention, improvements in living standards and better data. The amendments to the simplified grading system described here should help ensure that it continues to be useful for estimating the burden of trachoma in population-based surveys, and for evaluating the impact of control efforts over time.^19^
